# Combined measurement of perfusion, venous oxygen saturation, and skeletal muscle T_2_* during reactive hyperemia in the leg

**DOI:** 10.1186/1532-429X-15-70

**Published:** 2013-08-19

**Authors:** Erin K Englund, Michael C Langham, Cheng Li, Zachary B Rodgers, Thomas F Floyd, Emile R Mohler, Felix W Wehrli

**Affiliations:** 1 Department of Radiology, Laboratory of Structural NMR Imaging, University of Pennsylvania Medical Center, 3400 Spruce Street, Philadelphia, PA 19104, USA; 2 Department of Anesthesiology, Stony Brook University Medical Center, Stony Brook, NY 11794, USA; 3 Department of Medicine, University of Pennsylvania School of Medicine, Philadelphia, PA 19104, USA

**Keywords:** Peripheral artery disease, Atherosclerosis, Microvascular function, Perfusion, Dynamic oximetry, T2*, BOLD, Reactive hyperemia, Skeletal muscle

## Abstract

**Background:**

The function of the peripheral microvascular may be interrogated by measuring perfusion, tissue oxygen concentration, or venous oxygen saturation (SvO_2_) recovery dynamics following induced ischemia. The purpose of this work is to develop and evaluate a magnetic resonance (MR) technique for simultaneous measurement of perfusion, SvO_2_, and skeletal muscle T_2_*.

**Methods:**

Perfusion, Intravascular Venous Oxygen saturation, and T_2_* (PIVOT) is comprised of interleaved pulsed arterial spin labeling (PASL) and multi-echo gradient-recalled echo (GRE) sequences. During the PASL post-labeling delay, images are acquired with a multi-echo GRE to quantify SvO_2_ and T_2_* at a downstream slice location. Thus time-courses of perfusion, SvO_2_, and T_2_* are quantified simultaneously within a single scan. The new sequence was compared to separately measured PASL or multi-echo GRE data during reactive hyperemia in five young healthy subjects. To explore the impairment present in peripheral artery disease patients, five patients were evaluated with PIVOT.

**Results:**

Comparison of PIVOT-derived data to the standard techniques shows that there was no significant bias in any of the time-course-derived metrics. Preliminary data show that PAD patients exhibited alterations in perfusion, SvO_2_, and T_2_* time-courses compared to young healthy subjects.

**Conclusion:**

Simultaneous quantification of perfusion, SvO_2_, and T_2_* is possible with PIVOT. Kinetics of perfusion, SvO_2_, and T_2_* during reactive hyperemia may help to provide insight into the function of the peripheral microvasculature in patients with PAD.

## Background

Peripheral artery disease (PAD), a common manifestation of atherosclerosis in the lower limbs, causes significant morbidity and mortality in the United States [1-4]. Atherosclerotic plaques tend to develop at branch points in the peripheral arteries, increasing vascular resistance and limiting blood flow in the affected arteries [5]. Baseline blood flow to skeletal muscle is generally maintained through the recruitment of collateral arteries [5,6], however the vasculature is unable to quickly respond to changes in metabolic demand, such as those that occur with exercise or following a period of ischemia. Analogous to cardiac stress testing, the functional integrity of the peripheral vasculature can be interrogated by measuring the dynamic response to a period of induced ischemia.

Skeletal muscle can be stressed by means of an ischemia-reperfusion paradigm, which induces reactive hyperemia. Suprasystolic pressure applied by a cuff secured around the thigh halts distal blood flow for several minutes. Oxygen extraction in the stagnant capillary blood continues until it reaches a steady state, approximately 140 seconds into the period of arterial occlusion [7]. When pressure in the cuff is released, reactive hyperemia ensues with a surge in arterial flow resulting in an increase in perfusion [8] and oxygen concentration at the level of the capillary bed [7,9]. Additionally, venous oxygen saturation (SvO_2_) within the large draining veins sharply decreases as the desaturated blood formerly trapped in the capillary bed enters. As the tissue oxygenation recovers, SvO_2_ rises and eventually surpasses its baseline value during the time that oxygen delivery exceeds the oxygen extraction rate [10], or during which time physiologic shunting may be occurring.

Several magnetic resonance (MR) techniques can non-invasively evaluate the hyperemic response. Arterial spin labeling (ASL) is a well-known method to investigate perfusion in many vascular territories [11-16]. Skeletal muscle perfusion is an important parameter that quantifies microvascular blood flow thereby providing information on delivery of oxygen and nutrients to tissue. It has been shown that perfusion dynamics during reactive hyperemia are altered in PAD [17]. These findings correlate with both disease presence and severity.

A relative measure of tissue oxygenation can be obtained by measuring changes in the apparent transverse relaxation rate (T_2_*), also known as ‘blood oxygen-level dependent’ (BOLD) signal [18]. T_2_* can be measured using a multi-echo gradient-recalled echo (GRE) sequence. BOLD imaging has been extensively applied for functional activation studies in the brain [19], and can also provide information regarding activation and oxygenation of many other tissues including the kidneys [20] and skeletal muscle [7,8,21-23].

Dynamic measurement of SvO_2_ provides information about oxygen utilization in tissue. When continuously measured throughout an ischemia-reperfusion paradigm, intravascular blood can act as an endogenous tracer, allowing SvO_2_ time-course kinetics to provide information on endothelial function and vascular reactivity [10]. SvO_2_ can be dynamically measured using MR susceptometry [24]. Each of the aforementioned MR methods will be discussed in detail in the Theory section.

The kinetics of the hyperemic response can provide information on microvascular integrity and endothelial function. Healthy subjects are able to rapidly respond to increases in oxygen demand, and recover back to baseline more quickly than patients with PAD [17,25,26]. While methods for simultaneous quantification of perfusion and T_2_* or other markers of tissue oxygenation have been implemented [27,28], none have been able to investigate SvO_2_ time-course kinetics as well. Recovery dynamics are altered in each of these parameters in situations of impaired vascular function, as in PAD; therefore there is potentially added benefit to concurrent measurement. In this work, we developed a method to measure perfusion, SvO_2_, and T_2_* simultaneously. Such a technique allows for a full functional assessment of the peripheral vasculature during a single scan providing information on the temporal relationships between these various functional parameters.

## Theory

### Pulsed arterial spin labeling perfusion imaging

Pulsed arterial spin labeling (PASL) MRI is a well-established method for noninvasive perfusion imaging [13,14,29]. In one PASL variant developed by Raynaud, et al. [16], termed Saturation Inversion Recovery (SATIR), slice-selective (SS) and non-selective (NS) inversion pulses are applied for the tag and control images, respectively. Every acquisition is followed by a slice-selective saturation pulse to ensure the same initial magnetization in each dynamic image.

The normalized difference between the tag and control images can be used to quantify perfusion in physiologic units of milliliters of blood, per minute, per 100 grams of tissue, as described in [16]. The resulting Bloch equations can be solved analytically for perfusion (f) if the longitudinal relaxation times (T_1_) of arterial blood and tissue are assumed to be equal:

(1) f=−λT⋅lnMSST−MNSTMSST+MNST⋅1−eTT1+1

where M_SS_ and M_NS_ are the signal intensities (SI) in the image acquired after SS and NS inversion, respectively, λ is the tissue partition coefficient (0.9 mL/g), and T is the post-labeling delay (PLD), defined as the time between the inversion pulse and readout. At 3T, T_1blood_ = 1664 ms [30] and T_1tissue_ = 1420 ms [31]. A numerical calculation based on actual values for blood and tissue T_1_ shows that for perfusion values up to 100 mL/min/100g, using T_1tissue_ ≈ T_1blood_ = 1420 ms, underestimates perfusion by less than 5%.

SATIR has been successfully applied for perfusion imaging in the leg in healthy individuals [16], and the sequence has been employed to simultaneously measure BOLD [28,32], however it has not been integrated with a multi-echo GRE for venous oxygen saturation and BOLD measurements.

### MR susceptometry-based dynamic oximetry

MR susceptometry-based oximetry is a recently developed method for quantifying SvO_2_, measured in units of percent-oxygenated hemoglobin (%HbO_2_) [10,24,33,34]. Because deoxyhemoglobin is paramagnetic, a magnetic susceptibility difference exists between deoxygenated blood and oxygenated blood or tissue. This susceptibility difference induces a local magnetic field ∆*B* in the draining vein relative to the tissue, proportional to (1- SvO_2_/100). The incremental field ∆*B* can be determined by subtracting the phase accumulation of the MR signal in surrounding tissue from that inside the vein (∆*φ*). The phase is measured from successive echoes separated in echo time by ΔTE. By modeling the vein as a long paramagnetic cylinder it is possible to quantify intravascular SvO_2_ as:

(2) SvO2=1−2ΔφΔTEγΔχdo⋅Hct⋅B0cos2θ−13×100

where Δχ_*do*_ represents the susceptibility difference between fully oxygenated and fully deoxygenated blood (Δχ_*do*_ = 4π•0.27 ppm (SI units) [35,36]), B_0_ is the main magnetic field strength, and θ is the angle of the vessel with respect to B_0_[24,33]. It has been shown that for small angles, the induced field outside of the vessel is approximately homogeneous and independent of the susceptibility difference between the vein and tissue [37]. Therefore SvO_2_ can be measured using a field-mapping sequence, such as a multi-echo GRE. With this technique, one can quickly, directly, and noninvasively quantify intravascular SvO_2_ at high-temporal resolution.

### Skeletal muscle BOLD

In the microvasculature, as in the large veins, the paramagnetism of deoxyhemoglobin causes inhomogeneities in the local magnetic field. Because the microvessels are so small, it is not possible to directly measure the change in phase signal as is required for susceptometry-based oximetry. However, this local field perturbation also results in intravoxel phase dispersion, which causes the MR signal to decay more rapidly [18]. For instance, as oxygen saturation in the capillary bed decreases, the concentration of deoxygenated hemoglobin increases, resulting in an increased intravoxel phase dispersion thereby lowering the apparent transverse relaxation time, T_2_*. Thus changes in T_2_* in response to an ischemia-reperfusion paradigm can serve as a relative marker of tissue oxygenation [7]. The BOLD signal originates not only from changes in blood oxygen level, it is also sensitive to changes in perfusion, cellular pH, vessel diameter, and vessel orientation [9,21,23,38-40]. It has been postulated, however, that the BOLD signal changes primarily result from changes in the concentration of deoxyhemoglobin in the capillary bed [40]. Quantification of T_2_* can be achieved by fitting signal intensity data from a multi-echo GRE to a monoexponential function. Prior studies have shown the utility of investigating dynamic skeletal muscle BOLD during exercise [41-43], ischemia [38], reactive hyperemia [8], and in disease states [26,44].

### Simultaneous measurement of perfusion, SvO_2_, and T_2_*

**Figure 1:**
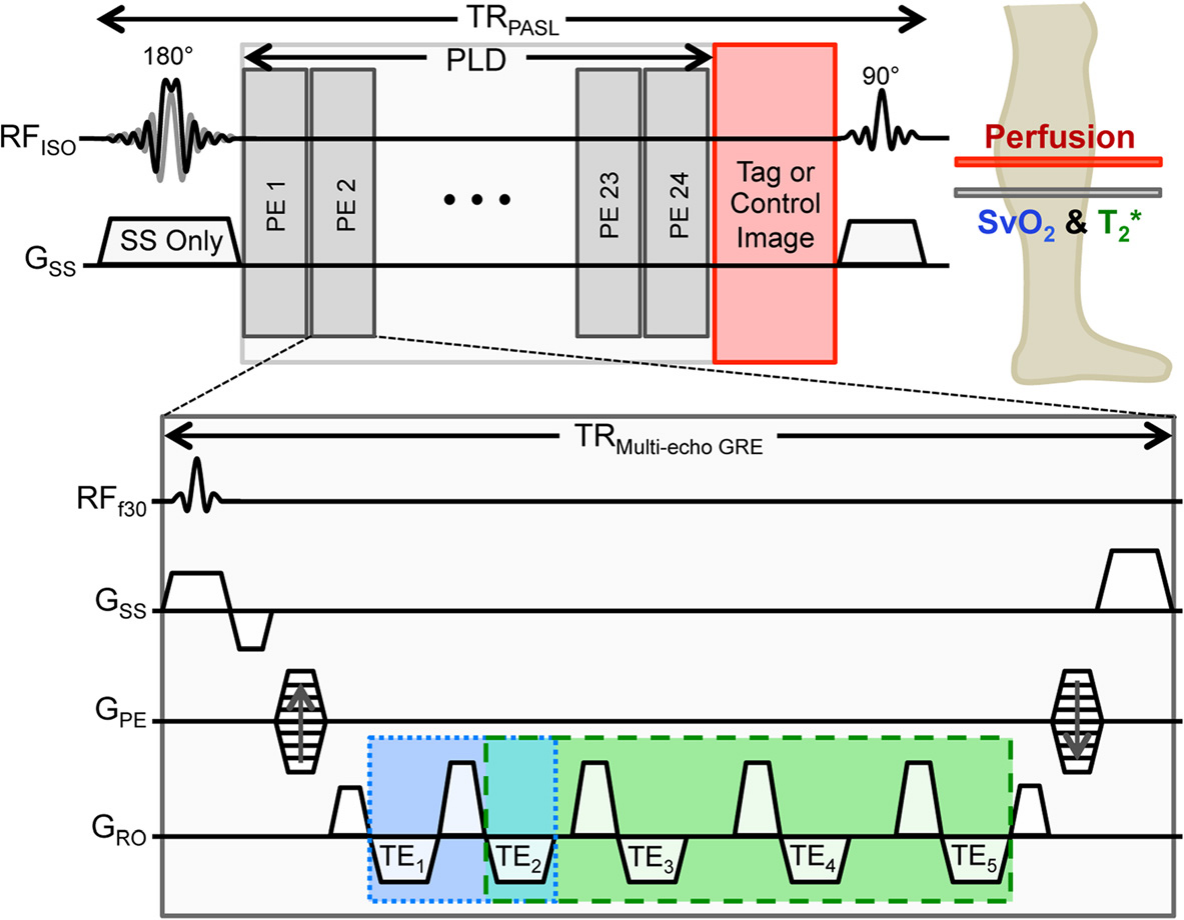
**Pulse sequence diagram of PIVOT.** A slice-selective (shown by SS only gradient) or non-selective adiabatic inversion pulse labels blood for perfusion imaging. During the PLD a keyhole multi-echo GRE acquires data downstream from the perfusion slice location for SvO_2_ (blue) and T_2_* (green) analysis. An EPI readout at isocenter (red) is used to acquire the images for perfusion quantification.

By interleaving a multi-echo GRE sequence in the PLD of a PASL sequence, perfusion, SvO_2_, and T_2_* can be concurrently measured. From multi-echo GRE data, the difference in signal phase between venous blood and surrounding tissue yields SvO_2_, while fitting the amplitude of the same data to a monoexponential function yields T_2_*. The sequence, termed Perfusion, Intravascular Venous Oxygen saturation, and T_2_* (PIVOT), makes use of the PLD dead time inherent to all PASL sequences to acquire SvO_2_ and T_2_* data at a separate slice location with a multi-echo GRE (Figure 1). This allows dynamic quantification of perfusion, SvO_2_, and T_2_* within a single scan.

In PIVOT, as in SATIR, tag and control conditions for perfusion imaging are achieved using SS and NS inversion pulses, respectively. During the PLD, a keyhole [45] multi-echo GRE sequence acquires data at a distal slice for SvO_2_ and T_2_* quantification. The distal location was chosen to ensure that the multi-echo GRE interleave does not impact the signal from previously labeled perfusing blood. Because the NS inversion affects both the PASL and multi-echo GRE slices, only multi-echo GRE data acquired following SS inversion are analyzed, though the interleave is run every PLD to control for magnetization transfer effects. The multi-echo GRE is immediately followed by a GRE-EPI readout at isocenter to capture data for perfusion quantification.

## Methods

### Study design

The University of Pennsylvania’s Institutional Review Board approved all imaging procedures, and each subject provided informed consent prior to his or her participation. To evaluate PIVOT compared to the standard measurement methods, five young healthy male subjects (27 ± 2 years old) were recruited and imaged on two separate occasions (Visit 1 and Visit 2). Four ten-minute scans were run in both sessions, each scan consisting of one minute baseline, three minute arterial occlusion, and six minute recovery. PIVOT, a repeat of the PIVOT scan (PIVOT Repeat), an otherwise identical PASL-only sequence, or otherwise identical multi-echo GRE-only sequence were run in a randomized order. To ensure the PASL interleave did not impact quantification of SvO_2_ and T_2_*, dynamic SvO_2_ and T_2_* results obtained with PIVOT were compared to the multi-echo GRE-only derived SvO_2_ and T_2_* results. Similarly, to ensure the multi-echo GRE interleave did not confound perfusion, the perfusion results obtained with PIVOT were compared to the PASL-only derived perfusion data. PIVOT was repeated to provide data on the intra-session variability of these parameters.

In addition, five PAD patients (67.2 ± 6.8 years old, ankle-brachial index (ABI) = 0.61 ± 0.14, 3 male) were drawn from an ongoing study and PIVOT imaging was performed during a single ischemia-reperfusion paradigm. For experiments in PAD patients, the total scan time was 12 minutes, with 2 minutes of baseline, 5 minutes of arterial occlusion, and 6 minutes of recovery. Since repeated arterial occlusions were not performed in PAD patients, a longer ischemic duration was used to ensure a maximal hyperemic response.

### Imaging

PIVOT, PASL-only, and multi-echo GRE-only sequences were written in SequenceTree [46] and exported for use on a 3T scanner (Siemens Medical Equipment; Erlanger, Germany). Each subject was positioned with the maximum girth of the calf centered in an 8-channel transmit/receive knee coil (Invivo, Inc; Gainesville, FL). For proximal arterial occlusion, a cuff was secured around the thigh and was rapidly inflated to 75 mmHg above the systolic pressure using the Hokanson E20 AG101 Rapid Cuff Inflation System (D. E. Hokanson, Inc; Bellevue, WA). Perfusion images were acquired with a partial Fourier GRE-EPI readout with the following parameters: FOV = 250 × 250 mm^2^; acquired matrix = 80×50, reconstructed to 80 × 80; slice thickness = 1 cm; slice location = isocenter; TR/TE = 1 s/8.05 ms; PLD = 952 ms. The keyhole multi-echo GRE used the following parameters: FOV = 96 × 96 mm^2^; keyhole acquired matrix = 96 × 24, (for SvO_2_ data analysis, reconstructed matrix = 96 × 96 using a fully sampled reference image obtained immediately after the dynamic PIVOT or multi-echo GRE acquisition; only dynamic data were used for T_2_* analysis); slice thickness = 1 cm; slice location = 3 cm inferior from isocenter; TR/TE_1_/TE_2_/TE_3_/TE_4_/TE_5_ = 38.12/3.78/6.99/12.32/19.32/26.32 ms. Perfusion, SvO_2_, and T_2_* each were quantified with two-second temporal resolution.

### Data analysis

**Figure 2:**
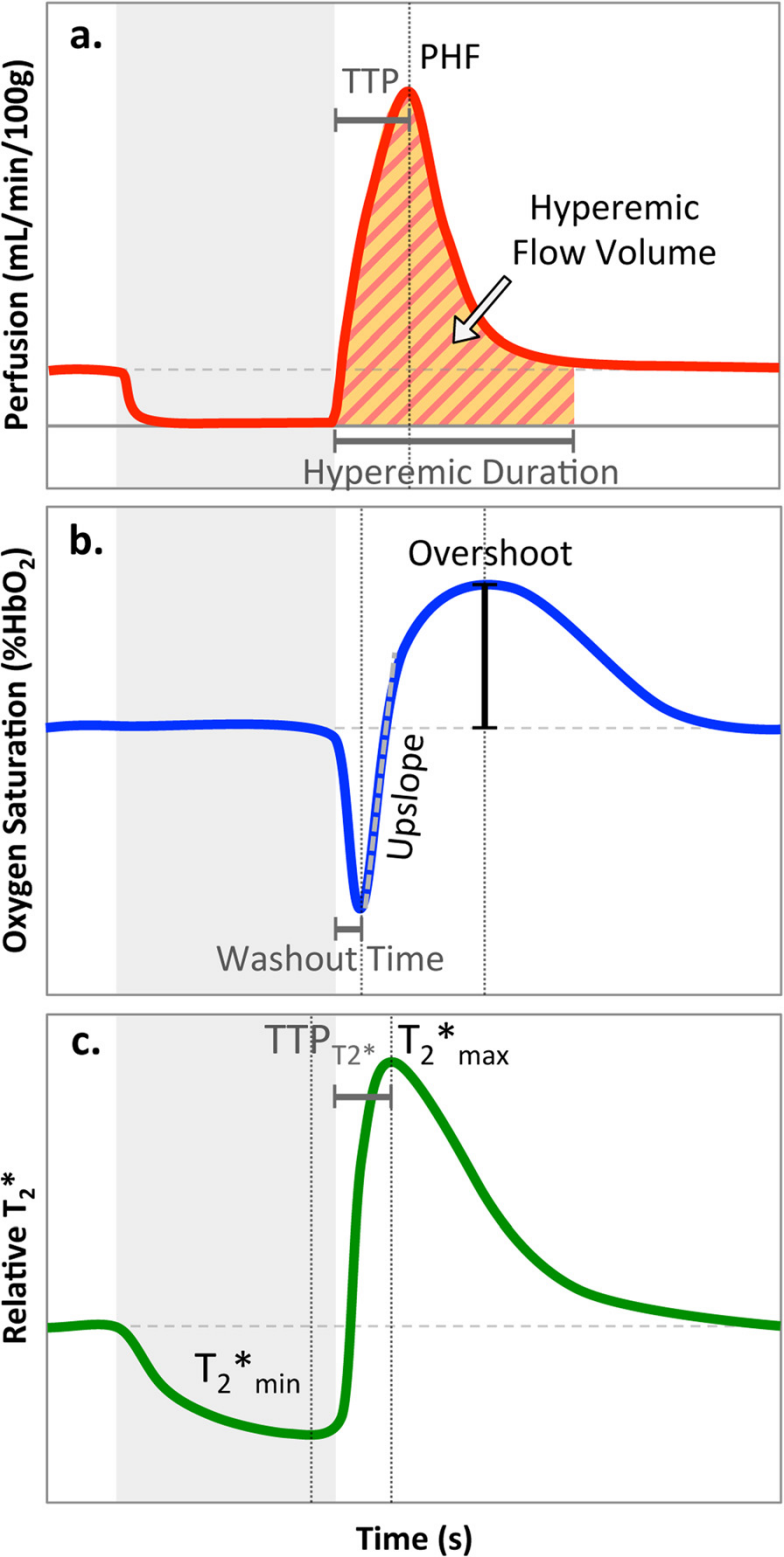
**Schematic of time-course for perfusion (a), SvO**_**2 **_**(b), and T**_2_* (c) illustrating the time-**course**-**derived metrics for each parameter.** Grey box indicates the period of proximal arterial occlusion.

*Perfusion*: Perfusion was measured in the soleus muscle. High spatial-resolution scout images were used as a reference, and a region of interest (ROI) in the soleus was visually selected on the EPI images. As the GRE-EPI data are inherently T_2_*-weighted, SI variation occurred throughout the ischemia-reperfusion paradigm due to the BOLD effect. Direct subtraction between adjacent NS and SS images would yield data with a mixture of perfusion, and ΔT_2_*-weighting. To account for this potential confound, NS time-series data were linearly interpolated to temporally match the SS time-course prior to perfusion quantification [47]. Perfusion was calculated using Equation 1 [16]. In order to correct for baseline perfusion offset, the average perfusion during the period of arterial occlusion was calculated and subtracted from each time-point as described in [47]. Peak hyperemic flow (PHF), time to peak (TTP), hyperemic flow volume (HFV), and hyperemic duration were measured (Figure 2a).

*Oximetry*: Dynamic SvO_2_ images were reconstructed to a matrix size of 96 × 96 using outer k-space data from a fully sampled reference image acquired immediately after the dynamic scan [45]. A phase difference image was generated for each of the dynamic time-points and the low spatial-frequency phase modulation was removed as described in [48]. An ROI was prescribed in the larger of the peroneal veins, and reference tissue was selected in an ROI immediately surrounding the peroneal vein. The phase accumulation was calculated from echoes at TE_1_ and TE_2_, with ΔTE = 3.21 ms and the difference between the intravascular and extravascular phase accumulation (∆*φ*) was computed. SvO_2_ was calculated from equation 2 [24]. Hematocrit of 0.45 was assumed for the healthy subjects, and in the PAD patients hematocrit was measured by blood draw. Washout time (time at which minimum SvO_2_ occurs), upslope, representing the rate of venous resaturation (maximum slope during recovery), and overshoot (peak SvO_2_ – baseline SvO_2_) were recorded (Figure 2b).

*T*_2_*: T_2_* analysis was performed on keyhole-only data since high spatial resolution is not as critical (acquired matrix = reconstructed matrix = 96 × 24). T_2_* was calculated by fitting a monoexponential function to magnitude SI from echoes TE_2_-TE_5_. Even though TE_1_ should have highest SNR, TE_1_ was not included in the monoexponential fitting because large switching gradients just prior to TE_1_ induced significant eddy current effects that would potentially confound T_2_* quantification. For BOLD analysis, average SI in an ROI prescribed in the soleus muscle for each of the four echoes was fitted to a mono-exponential function to determine T_2_* at each time-point. T_2_* values were normalized to the baseline average and relative T_2_*_min_, relative T_2_*_max_, and time to peak (TTP_T2*_) were determined (Figure 2c).

### Statistical analysis

The average and standard deviation of every time-point across all subjects and both sessions was calculated for PIVOT, PASL, and multi-echo GRE time-course data. Pearson’s correlation coefficient was calculated to compare the time-courses measured with PIVOT and the standard methods.

For each key time-course-derived parameter, Wilcoxon signed-rank tests were used to assess whether statistically significant differences exist between PIVOT and the standard method. Specifically, PIVOT-derived perfusion parameters were compared to results obtained with PASL-only, and PIVOT-derived SvO_2_ or T_2_* parameters were compared to results from the multi-echo GRE-only scan. Wilcoxon signed-rank tests were also used to determine whether significant differences exist between key parameters measured with PIVOT and PIVOT Repeat. Wilcoxon signed-rank tests were used in lieu of a standard paired Student’s *t*-test as only five subjects were enrolled in the evaluation study and thus it cannot be assumed that the data are normally distributed. Statistical significance was set at p < 0.05.

To assess both intra-session and inter-session repeatability, the average within-subject coefficient of variation (CV) was calculated. Specifically, to calculate intra-session repeatability, for each parameter the within-subject standard deviation across PIVOT and PIVOT Repeat from Visit 1 was averaged across subjects, then divided by the between-subject mean parameter value from Visit 1. The same analysis was performed for intra-session repeatability on data acquired during Visit 2. Similarly, to calculate inter-session repeatability, the within-subject standard deviation across all PIVOT scans from Visit 1 and Visit 2 (4 measurements per subject) was averaged across all subjects and divided by the between-subject mean parameter value from both visits.

In the PAD patients, all time-course parameters described above were calculated, but no statistical analyses were performed since patients included in this preliminary study have varying disease severity and the ischemia-reperfusion paradigm was slightly different. The purpose of including PAD patient data was for proof of principle and to explore differences that exist between patients and healthy subjects.

## Results

### PIVOT evaluation in young healthy subjects

**Figure 3:**
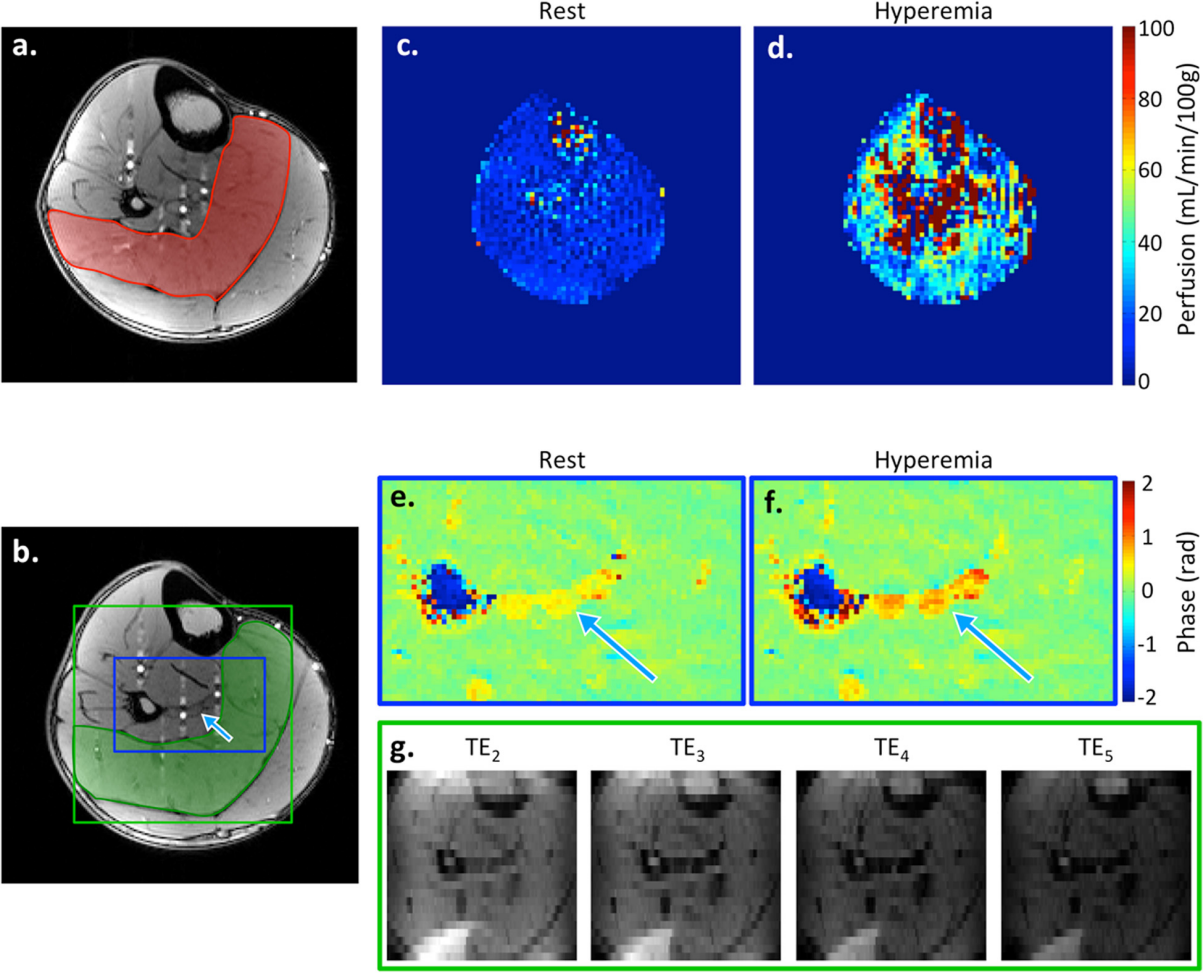
**Example images from a representative young healthy subject.** High-resolution scout images located at isocenter **(a)** and 3 cm inferior **(b)**, corresponding to the PASL and multi-echo GRE slice locations, respectively. The soleus is indicated in red **(a)** and green **(b)**, and the blue arrow points to the peroneal vein. Perfusion images represent baseline **(c)** and peak hyperemic flow **(d)**. Phase images are shown for baseline **(e)** and the washout time **(f)**. Note the increased phase accrual in the three veins at washout time, corresponding to a decrease in SvO_2_. The blue arrow identifies the peroneal vein that was used for dynamic SvO_2_ analysis. Multi-echo GRE magnitude images for each of the echo times used to quantify T_2_* are shown in **(g)**.

Example images are shown for a representative subject in Figure 3. High-resolution images corresponding to the PASL (isocenter) and multi-echo GRE slices (3 cm inferior) along with highlighted regions indicating the muscle or vein of interest are included in panels (a) and (b). Sample baseline and peak hyperemia perfusion maps are shown in (c) and (d), respectively. These images highlight the dramatic increase in perfusion that occurs in response to induced ischemia. The green box in (b) shows the full FOV of the multi-echo GRE. Because the FOV of the multi-echo GRE was only 96 × 96 mm, aliasing along the phase-encoding direction occurred in several subjects. In this subject, the tibialis anterior muscle has wrapped posteriorly, and part of the gastrocnemius muscle has wrapped anteriorly. This aliasing did not affect the quantification of SvO_2_, and wrapped regions were avoided when selecting the soleus ROI for T_2_* measurement. Sample phase images used for SvO_2_ quantification at baseline and hyperemia (corresponding to the minimum SvO_2_, which occurs at the washout time) are shown in (e) and (f). Keyhole reconstruction was used for the phase images to achieve higher apparent in-plane spatial resolution (1 × 1 mm), which is necessary in order to resolve the veins. However, since spatial resolution is less critical for T_2_* only data acquired every TR was used for analysis. Thus each image in (g) has in-plane resolution of 1 × 4 mm.

**Figure 4:**
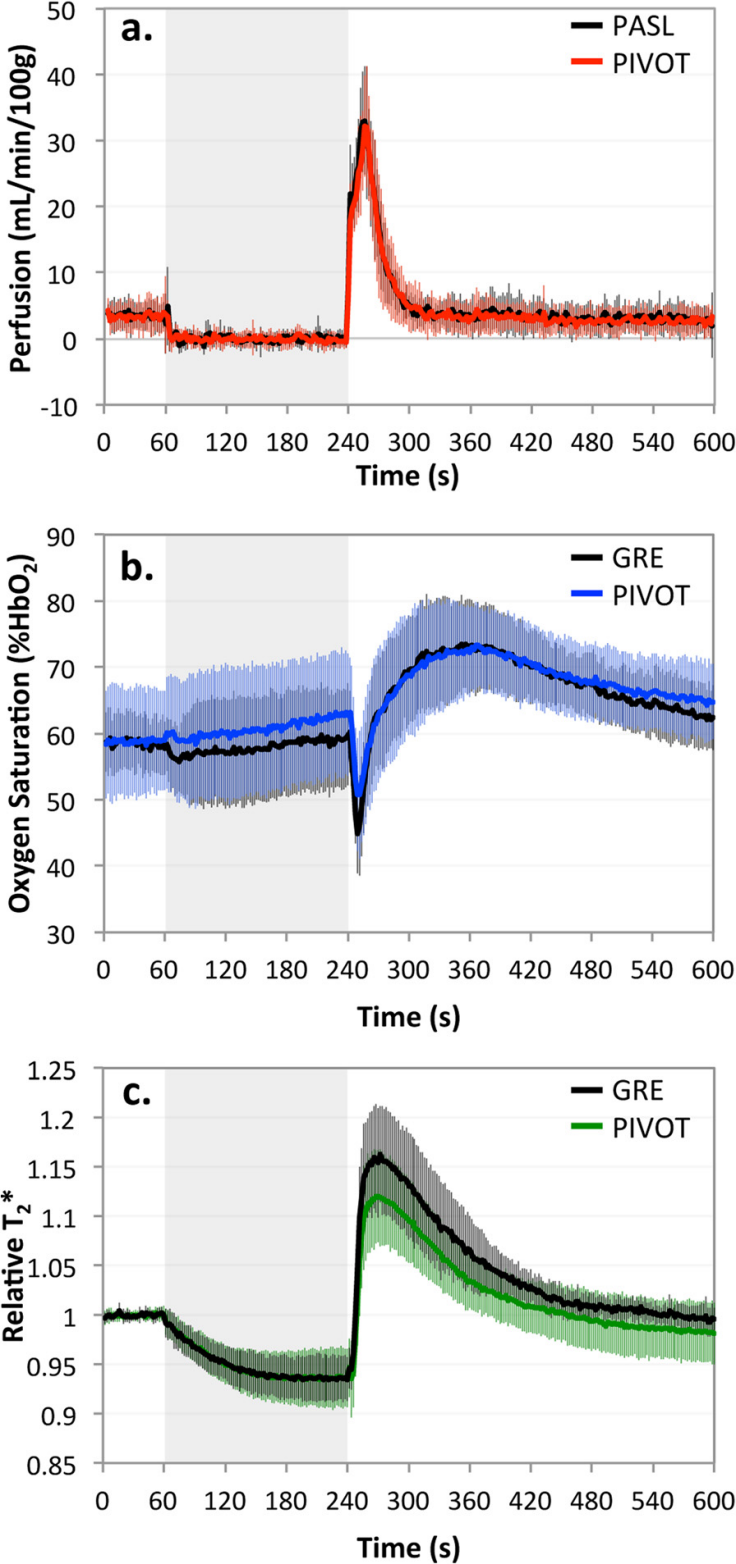
**Average time**-**course data measured with PIVOT and standard measurement methods. (a)** Average perfusion time-course across all young healthy subjects measured with PIVOT (red) and PASL (black). Average SvO_2_**(b)** and T_2_* **(c)** time-courses measured with PIVOT (blue, green, respectively) and a multi-echo GRE (black). Error bars indicate standard deviation. Grey box indicates period of arterial occlusion.

Data for all healthy subjects were averaged to yield an average perfusion, SvO_2_, or T_2_* time-course in order to investigate the correlation of the results between PIVOT and PASL-only or multi-echo GRE-only methods. For each parameter, average and standard deviation of the time-courses across all subjects is shown in Figure 4. Following cessation of arterial occlusion, the typical reactive hyperemia response is seen in each of the measured parameters. The time-course measured with PIVOT is in good agreement with PASL-only or multi-echo GRE-only-measured responses. The correlation coefficient between PIVOT and PASL average perfusion time-course is 0.99, and between PIVOT and multi-echo GRE average SvO_2_ and T_2_* time-courses are 0.98 and 0.99, respectively.

Average (standard deviation) of key time-course parameters from PIVOT and the standard measurement methods are shown in Table 1. The Wilcoxon signed-rank tests did not detect statistically significant differences between PIVOT and PIVOT Repeat, or between PIVOT and the standard measurement method for any of the key time-course parameters (p > 0.05). Table 2 summarizes the intra-session and inter-session repeatability measured with PIVOT.

**Table 1:** **Means and standard deviations** (**in parentheses**) **of key time**-**course metrics measured with PIVOT and standard measurement methods in five young healthy subjects on two separate occasions**

	***Visit 1***			***Visit 2***		
	**PIVOT**	**PIVOT Repeat**	**Standard Method**	**PIVOT**	**PIVOT Repeat**	**Standard Method**
***Perfusion*** (***Standard Method*** = ***PASL***)						
PHF (mL/min/100g)	34.8 (7.5)	34.5 (10.2)	37.9 (9.0)	39.2 (4.1)	38.3 (6.0)	37.6 (5.6)
TTP (s)	19.6 (3.6)	18.0 (3.5)	17.2 (2.3)	18.4 (5.7)	17.2 (4.1)	17.6 (5.2)
HFV (mL/100g)	17.0 (5.3)	14.0 (5.0)	16.2 (5.6)	16.7 (5.4)	14.4 (1.9)	16.1 (2.3)
Hyperemic duration (s)	53.6 (15.7)	46.8 (13.8)	53.6 (10.4)	48.8 (14.9)	44.8 (10.3)	49.6 (14.2)
***SvO***_***2***_ (***Standard Method*** = ***multi***-***echo GRE***)						
Washout time (s)	11.6 (2.2)	10.0 (2.0)	10.4 (1.7)	12.4 (3.6)	11.2 (4.8)	10.0 (1.4)
Upslope (%HbO_2_/s)	0.83 (0.32)	1.08 (0.47)	1.21 (0.54)	1.14 (0.42)	0.91 (0.43)	1.13 (0.48)
Overshoot (%HbO_2_)	17.6 (7.5)	17.1 (6.1)	16.8 (7.1)	18.6 (6.5)	16.5 (7.6)	18.3 (5.0)
***T***_2_* (***Standard Method*** = ***multi***-***echo GRE***)						
Baseline T_2_* (ms)	23.4 (1.4)	22.5 (1.5)	23.1 (2.0)	22.0 (1.9)	21.7 (1.6)	22.2 (2.0)
Relative T_2_*_min_	0.90 (0.04)	0.93 (0.03)	0.92 (0.01)	0.92 (0.03)	0.93 (0.04)	0.94 (0.02)
Relative T_2_*_max_	1.09 (0.03)	1.13 (0.04)	1.16 (0.05)	1.14 (0.05)	1.15 (0.05)	1.18 (0.05)
TTP_T2*_ (s)	34.0 (11.6)	29.2 (3.6)	26.8 (5.2)	28.4 (3.6)	24.8 (5.0)	28.4 (4.8)

No statistically significant differences were detected in any measured parameter.

**Table 2:** **Summary of intra**-**session and inter**-**session repeatability for all time**-**course**-**derived metrics is presented as the within-subject coefficient of variation**

	**Intra**-**session**	**Intra**-**session**	**Inter**-**session**
	**Visit 1**	**Visit 2**	**Visit 1 vs**. **2**
***Perfusion***			
PHF (mL/min/100g)	6.3%	5.0%	12.4%
TTP (s)	6.0%	11.1%	16.4%
HFV (mL/100g)	18.8%	23.4%	18.9%
Hyperemic duration (s)	15.2%	25.4%	19.9%
***SvO***_***2***_			
Washout time (s)	15.7%	12.0 %	18.9%
Upslope (%HbO_2_/s)	26.2%	15.8%	37.3%
Overshoot (%HbO_2_)	15.3%	18.3%	6.5%
***T***_2_*			
Baseline T_2_* (ms)	3.1%	1.3%	4.6%
Relative T_2_*_min_	2.4%	0.9%	1.8%
Relative T_2_*_max_	3.0%	1.1%	3.9%
TTP_T2*_ (s)	17.9%	9.6%	19.9%

### PIVOT in PAD patients

**Figure 5:**
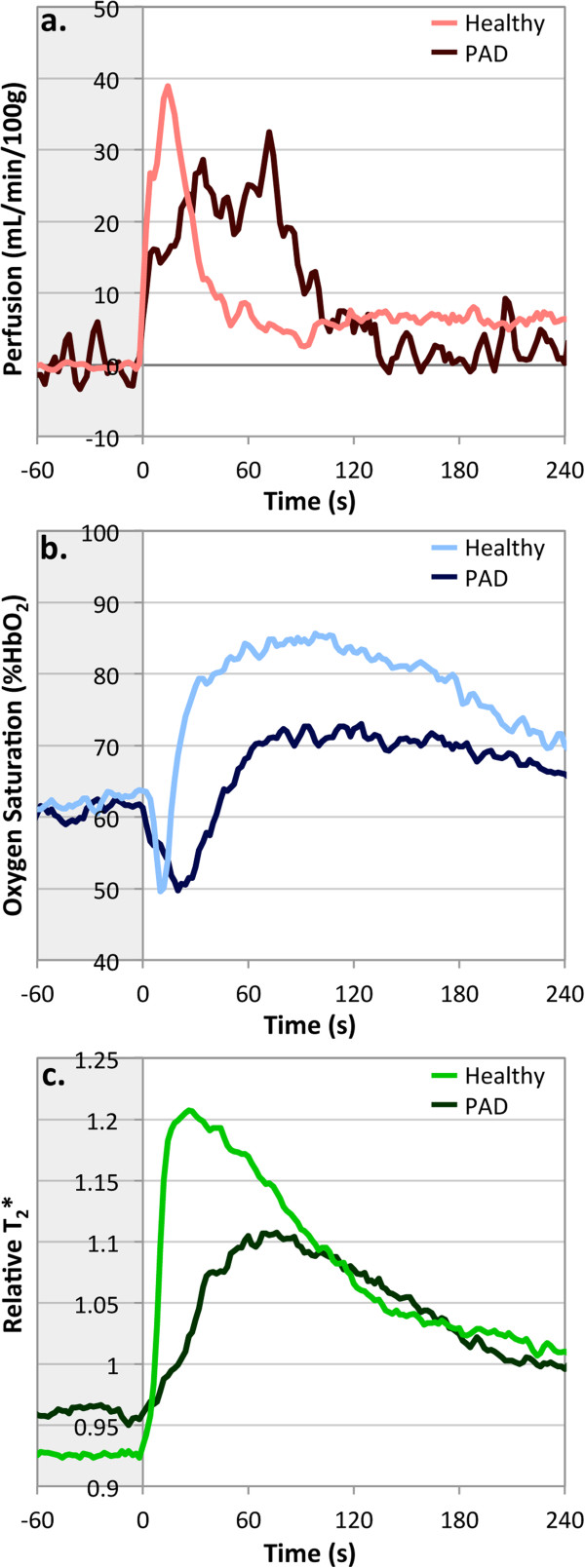
**Time**-**courses measured with PIVOT for perfusion (a), SvO**_**2 **_**(b), and T**_2_* (c) in one representative young healthy subjects and one PAD patient (PAD #5 in Table2**).** Light colored lines represent healthy subject, and dark colored lines represent PAD patient. Grey box indicates period of arterial occlusion. PAD patient exhibits a blunted and delayed hyperemic response for each of the measured parameters compared to the young healthy subject.

Figure 5 shows the reactive hyperemia time-courses for perfusion, SvO_2_, and T_2_* measured with PIVOT in a single PAD patient and a representative young healthy subject. A summary of key time-course parameters measured in individual PAD patients is presented in Table 3 along with average values for young healthy subjects. The perfusion time-course data show that patients experienced a lower PHF, a delay in TTP, a prolonged hyperemic duration, and a greater HFV. The SvO_2_ response was also delayed and blunted; PAD patients exhibited a longer washout time, and reduced upslope and overshoot. T_2_* data showed characteristic changes expected in patients with reduced endothelial function. PAD patients had higher T_2_*_min_, even though the ischemic duration is 5 minutes instead of 3 minutes as in the healthy subjects. Patients’ T_2_*_max_ was lower and TTP_T2*_ was delayed. These results are in agreement with previous findings measuring perfusion [17], SvO_2_[10,25], or T_2_* [26,44] individually in PAD patients.

**Table 3:** PIVOT results in individual PAD patients and for the average of all young healthy subjects

	**Healthy**	**PAD** #**1**	**PAD** #**2**	**PAD** #**3**	**PAD** #**4**	**PAD** #**5**
ABI	N/A	0.41	0.52	0.68	0.72	0.74
***Perfusion***						
PHF (mL/min/100g)	37.0 (6.1)	26.9	22.0	31.3	29.9	37.3
TTP (s)	19.0 (4.5)	110	72	90	52	32
HFV (mL/100g)	16.9 (5.1)	48.2	26.5	78.7	37.7	33.8
Hyperemic duration (s)	51.2 (14.6)	182	124	240	110	108
***SvO***_***2***_						
Washout time (s)	12.2 (2.2)	48	50	22	36	22
Upslope (%HbO_2_/s)	1.0 (0.6)	0.24	0.18	0.45	0.69	0.45
Overshoot (%HbO_2_)	18.1 (6.5)	16.4	17.9	13.2	16.7	12.9
***T***_2_*						
Baseline T_2_* (ms)	22.6 (1.7)	22.2	22.4	21.6	20.4	20.0
Relative T_2_*_min_	0.91 (0.03)	0.93	0.94	0.92	0.95	0.94
Relative T_2_*_max_	1.11 (0.04)	1.04	1.04	1.11	1.04	1.12
TTP_T2*_ (s)	32.8 (8.8)	120	60	94	56	68

## Discussion

### PIVOT repeatability

The repeatability assessment comparing perfusion, SvO_2_, and T_2_* metrics derived from the two successive PIVOT scans show some intra-session variability. This variability could be physiologic in nature or could be due to noise in the time-course data. Given the low average baseline signal standard deviation (perfusion: 1.6 mL/min/100g; SvO_2_: 1.5 %HbO_2_; T_2_*: 0.5%) it is likely that intra-session variations of key measured parameters are outweighed by physiologic variability during separate ischemia-reperfusion episodes. In all cases there were no significant differences detected between time-course parameters measured with PIVOT and PIVOT Repeat (p>0.05), suggesting that there was no training effect due to multiple periods of ischemia. This finding justifies the comparison of within-session PIVOT to PASL-only or to multi-echo GRE-only sequences, even though the data were acquired separately.

### PIVOT effect on perfusion quantification

In comparing perfusion metrics measured with PIVOT to those measured with an otherwise identical PASL-only sequence, it is evident that the two measurements provide similar values of all perfusion time-course-derived metrics. Specifically, there were no significant differences between any of the key parameters measured with PIVOT or with PASL. This suggests that the multi-echo GRE interleave does not impact the quantification of perfusion.

Perfusion time-course metrics had expected results for longitudinal reproducibility. A CV of approximately 20% has been reported in previous studies [15,49]. Perfusion varies physiologically with time of day [50], hydration level [51], and hormonal fluctuations [52] among other factors [53]. Care was taken to schedule Visits 1 and 2 at the same time, and all subjects were instructed to refrain from caffeine intake and vigorous activity for 12 hours prior. Yet even within a single scan there was variability in the hyperemic response from one ischemia-reperfusion episode to another, suggesting that these physiologic factors cannot be completely controlled. The time-course data averaged over all experiments and all healthy subjects showed good correlation in the shape and magnitude of the reactive hyperemia response, reflected by the high correlation coefficients. Thus not only were the key time-course-derived metrics not significantly different, but that the overall response was highly similar.

Peak hyperemic flow measured with either PIVOT or PASL was somewhat lower than previously reported values, where PHF was measured to be 50 ± 13 mL/min/100 g using the same SATIR perfusion preparation, but with a RARE readout [16]. PHF in the soleus in our study was lower, reaching only 37.0 ± 6.1 mL/min/100 g. This difference in perfusion could be attributed to the fact that Raynaud et al. quantified whole-leg perfusion [16], while individual muscle perfusion was calculated here. Additionally, care was taken to exclude vessels from the ROI, as their inclusion would increase measured perfusion. In another study by Proctor and colleagues, perfusion was measured in the calf of 64 men using strain gauge plethysmography [54], a technique considered to be a standard for limb perfusion measurement. Peak perfusion in the calf was found to be 35.1 ± 1.1 mL/min/100 g following a period of 10 minutes of ischemia. This work and that by Raynaud [16] and Proctor [54] report much lower PHF than a similar study by Wu et al. using continuous arterial spin labeling (CASL). In Wu’s study, PHF in the soleus muscle was found to be 116 ± 57 mL/min/100 g [15], however the temporal resolution in CASL was limited to 16 seconds. Using an EPI readout, the magnitude signal over the course of an ischemia reperfusion paradigm varies substantially due to the BOLD effect. If the time course is not sampled with high enough temporal resolution, the changing signal intensity due to the BOLD effect may contaminate the quantification of perfusion [55]. The higher temporal resolution data acquired with SATIR can track the changes in signal intensity better and thus may be less susceptible to BOLD contamination, allowing more accurate quantification of perfusion.

Time to peak perfusion agreed with Raynaud’s values [16], however it was much shorter than the TTP reported by Wu [15]. Again, this discrepancy between our work and that of Wu et al. could be due to the better temporal resolution of SATIR over CASL. The temporal resolution for PIVOT and SATIR was 2 seconds, while that of the CASL sequence employed in Wu’s work was 16 s. The improvement in temporal resolution was in part due to the pulsed tagging scheme used in SATIR, in which arterial tagging takes only 8 ms instead of 2 seconds as in CASL.

Hyperemic flow volume quantified the total blood delivery during hyperemia, and along with the hyperemic duration may provide more qualitative measures of hyperemia. HFV in particular may be less sensitive to time-course noise compared to PHF and TTP. PHF and TTP are determined based on a single data-point, whereas HFV is the total integrated area. The values we reported for HFV and hyperemic duration were lower and shorter than those reported by Wu, et al. [15], which is not surprising since our measured PHF and TTP are lower and shorter as well.

### PIVOT effect on SvO_2_ quantification

High spatial resolution is necessary to measure the phase in the vein; therefore the keyhole multi-echo GRE data was supplemented with outer k-space data from a fully-phase-encoded reference scan that was run at the end of the dynamic acquisition. Langham et al. have previously shown that keyhole reconstruction provides accurate SvO_2_ results with high temporal resolution during reactive hyperemia in the femoral vein [56].

No significant differences were detected between time-course metrics measured with PIVOT or with the multi-echo GRE. While inter-session variability was present, it was comparable to the intra-session variability measured between PIVOT and PIVOT Repeat on the same visit. Average washout time was slightly lower than previously reported in the femoral vein of young healthy subjects (17 ± 7 s), but upslope and overshoot were in agreement with prior results [10]. The lower washout time could be explained by the fact that the tourniquet system used in this study deflated much more quickly than that used in [10]. The resulting decrease in the resistance to arterial flow could potentially shorten the washout time. Another potential reason that washout time differs is that we investigated SvO_2_ in the peroneal vein, as opposed to the more superior femoral vein. Thus not only does the peroneal vein collect from a smaller volume of muscle, but the distance between the capillary bed and peroneal vein was also smaller. Overall, key parameters measured with PIVOT agree with multi-echo GRE-derived data, suggesting that inclusion of the PASL interleave does not impact quantification of SvO_2_.

### PIVOT effect on T_2_* quantification

In both PIVOT and multi-echo GRE T_2_* data, the signal intensity in the ROI was first averaged then fit to a mono-exponential function. The fitting of average signal intensity was nearly perfect with an overall average R^2^ for all T_2_* fits of 0.999.

During the period of arterial occlusion, relative T_2_* was found to decrease as the deoxyhemoglobin concentration in the capillary bed increases. Following cuff release, hyperemic arterial inflow replenishes blood in the capillary bed, bringing oxygenated arterial blood in and moving desaturated blood into the large draining veins. Relative T_2_* increased during reactive hyperemia due to the increase in perfusion and the decrease in deoxyhemoglobin in the capillary bed. Even though perfusion and T_2_* were measured at separate slice locations, they both represent changes that occur in the soleus muscle. TTP_T2*_ was longer than perfusion TTP, suggesting that the increase in oxygen concentration at the level of the capillary bed lasted longer than the increase in microvascular flow. This finding is in agreement with a prior study investigating the combined measurement of perfusion and BOLD during reactive hyperemia [28]. Duteil et al. suggest that because the brief period of arterial occlusion does not cause significant oxygen debt in muscle (as shown by [57]), the muscle’s demand for oxygen remains unchanged and thus the increase in perfusion results in a decrease of oxygen extraction, causing the BOLD signal to increase [28]. The decrease in oxygen extraction also physiologically manifests as the SvO_2_ overshoot.

The measured values are consistent with previous literature reported values for baseline T_2_* [58], relative T_2_*_min_[44], T_2_*_max_[26,59], and TTP_T2*_[26]. Comparisons between PIVOT and multi-echo GRE key time-course parameters yielded no significant differences, and the T_2_* time-course data measured with PIVOT and multi-echo GRE were highly correlated (Pearson’s r = 0.99). These results indicate that T_2_* quantification in a downstream slice with PIVOT is not affected by the PASL interleave.

### Considerations for applying PIVOT in PAD patient studies

Statistical comparisons between PAD patients and young healthy subjects were not made because the ischemic duration differed in the two cohorts. As repeated periods of arterial occlusion were used to compare PIVOT to standard measurement methods in healthy subjects, a shortened ischemic duration was used, allowing enough time for the four scans to be performed during one hour of scanning. PAD patients included in this preliminary evaluation had varying disease severity, represented by the diverse ABIs. A five-minute period of arterial occlusion was employed in PAD patients to ensure the maximal hyperemic stimulus. The PAD patient data were, however, included to explore the range of values that exist between states of health and disease. In agreement with the results of Wu et al., PAD patients exhibited a decrease in PHF and an increase in TTP [17]. SvO_2_ data showed a blunted and delayed response, in agreement with Langham et al. [10,25]. T_2_* data showed the characteristic alterations during the period of ischemia [44] and reactive hyperemia [26].

Quantification of multiple parameters may improve diagnosis and enhance power for detection of the response to therapeutic intervention. The traditional marker of disease severity in PAD is the ankle-brachial index (ABI), which is the ratio of systolic blood pressures measured at the level of the ankle and in the brachial artery. The ABI primarily represents occlusions and stenoses on the macrovascular level. Previous studies have shown that physiologic improvements such as increased peak walking time do not correlate with clinically significant changes in ABI [60,61]. PIVOT provides a measure of microvascular function, thus may be more sensitive to early treatment effects. By measuring many parameters, PIVOT will provide insight into the relationship between impairments in perfusion, SvO_2_, and T_2_*.

In this study we showed that no major measurement bias in key time-course parameters was introduced by using PIVOT instead of the standard individual measurement methods. However, it should be noted that the small sample size affects the power to detect such a bias. Even though no measurement bias was detected, the precision and thus statistical power of PIVOT-derived measures was limited by physiologic variability, which cannot be completely controlled. In order to use this method to assess disease presence or monitor a treatment effect, the study must be well designed, controlling for factors that are known to affect perfusion and the hyperemic response [50-53].

The preliminary PAD patient data showed that for many of the key time-course parameters there was a relatively wide range of values that exist between states of health and disease. A recent study performed at 1.5T showed relatively poor repeatability of BOLD measurements with the exception of TTP_T2*_ during reactive hyperemia in both healthy subjects and PAD patients [49]. In our study a lower inter-session CV, and hence better repeatability, was found for several key T_2_* parameters in healthy subjects. The improvement in repeatability may be due to the higher field strength, which confers increased signal to noise ratio and greater BOLD signal contrast. A longitudinal study will be necessary to determine the repeatability of PIVOT measures in PAD patients. Additionally vascular reactivity decreases with age [25], therefore it will be important to compare PIVOT results obtained for PAD patients to age-matched healthy controls to assess the impact of age on PIVOT-derived measures of microvascular function.

## Conclusions

In summary, we have introduced a quantitative MR method that measures perfusion, SvO_2_, and T_2_* simultaneously, thereby allowing a comprehensive assessment of the functional integrity of the peripheral microvasculature during a single ischemia-reperfusion paradigm. The added value of the proposed approach will require rigorous evaluation in cohorts of patients with impaired peripheral circulation in comparison to their healthy peers. In the future PIVOT could possibly serve as a means to monitor disease progression and effectiveness of intervention.

## Competing interests

The authors’ declare that they have no competing interests.

## Authors’ contributions

EE, EM, TF and FW conceived the study. EE and ZR analyzed and interpreted data. EE, ML, and CL worked on the development of the MR sequence and protocol. EE drafted the manuscript. All authors revised the manuscript critically for important intellectual content, read and approved the final manuscript.
